# Supplementary material for: Integrated analysis of the aqueous humor microbiome and lens capsule transcriptome in high myopia cataract: a pilot study
Source: Front Med (Lausanne). 2026 Jun 16;13:1845205. doi: 10.3389/fmed.2026.1845205 (PMC13314463; doi:10.3389/fmed.2026.1845205)
Supplement: Supplementary file 3 [file Data_Sheet_1.zip › 5.Association/indicator/C372089/indicator.pdf]

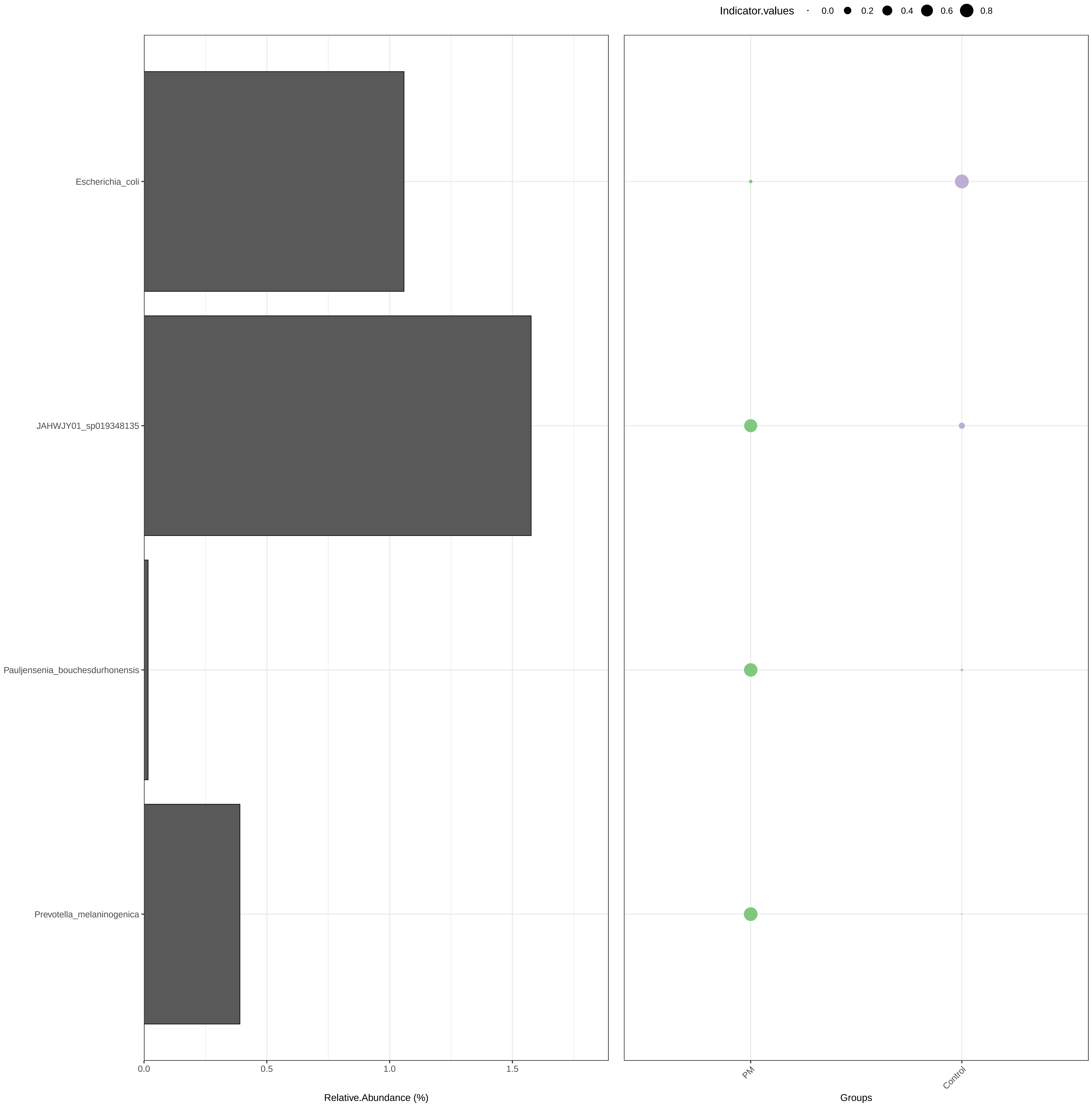

Indicator.values
0.0
0.2
0.4
0.6
0.8
Escherichia_coli
JAHWJY01_sp019348135
Pauljensenia_bouchesdurhonensis
Prevotella_melaninogenica
0.0
0.5
1.0
1.5
Relative.Abundance (%)
PM
Control
Groups
